# Merkel Cell Tumor: A Mystery Beneath the Surface

**DOI:** 10.7759/cureus.92164

**Published:** 2025-09-12

**Authors:** Axel R Marquez-Nuñez, Nataly C Berrezueta Córdova, Nishdaly A Rodríguez Valencia, Elisa Vega-Memije, Leticia Boeta Ángeles

**Affiliations:** 1 Department of Dermatology, National Autonomous University of Mexico, Mexico City, MEX; 2 Department of Dermatology, Hospital General "Dr. Manuel Gea González", Mexico City, MEX; 3 Department of Dermatology, Hospital Juárez del Centro, Mexico City, MEX

**Keywords:** cutaneous apudoma, merkel cell carcinoma, neuroendocrine carcinoma, skin malignancy, trabecular carcinoma of the skin

## Abstract

Merkel cell carcinoma (MCC) is an aggressive skin condition that requires prompt diagnosis and management. It predominantly affects older adults and has a high propensity for recurrence and metastasis. The acronym AEIOU, which stands for asymptomatic, expanding rapidly, immunosuppression, older than 50 years, and UV radiation, is often used in the approach to diagnosis.

We present a unique and intriguing case of a 70-year-old patient with a solitary and asymptomatic exophytic lesion on her right forearm that had been present for one year. She had not received any treatment or medical advice to date.

Our case contributes to the literature on MCC, highlighting the variety of differential diagnoses that can occur in an atypical variety such as the one we are describing. We recall the relevance of the histopathological approach, emphasizing the crucial role of immunohistochemistry in the presumptive diagnosis of this entity.

## Introduction

Merkel cell carcinoma (MCC) is a neuroendocrine carcinoma with high rates of recurrence and metastasis [1]. MCC was first described by Cyril Toker in 1972, who named it trabecular carcinoma based on its infiltrating trabecular-like growth pattern [2]. In 1985, its similarity to Merkel cells was recognized [3]. MCC carcinogenesis is associated with Merkel cell polyomavirus or chronic exposure to ultraviolet (UV) light, taking into account other factors that influence its development, such as advanced age and immunosuppression [4]. In the United States, the Surveillance, Epidemiology, and End Results (SEER) program reported 6,600 cases between 2000 and 2013, mentioning that the incidence increased from 0.5 cases per 100,000 in 2000 (95% CI: 0.4-0.5) to 0.7 per 100,000 in 2013 (95% CI: 0.7-0.8) [5].

Clinically, MCC presents as a solitary, painless, rapidly growing, skin-colored to purplish tumorous growth, primarily on the head and neck [4]. The acronym AEIOU, which stands for asymptomatic, rapidly expanding, immunosuppression, older age, and ultraviolet radiation, encircles the clinical characteristics of a suspected diagnosis [1,6]. This acronym has a sensitivity of 89% in patients with three of the five characteristics; however, it is not a specific tool [1], which is why a biopsy corroborates this presumptive diagnosis.

Histopathology often shows growth with a nest-like and trabecular pattern, although they may display overlapping features. Neoplastic cells may be oval and may be larger than mature lymphocytes. The cytoplasm is scant, with the presence of ovoid nuclei with dispersed chromatin. Mitotic figures are usually abundant [1,4]. MCC cells express various cytoskeletal keratin (CK) types I and II, particularly CK20. Neoplastic cells also express neuroendocrine markers such as synaptophysin, chromogranin, somatostatin, calcitonin, and vasoactive intestinal peptide [1,4]. The primary differential diagnoses for this entity are squamous cell carcinoma, adnexal tumors, and amelanotic melanoma [4].

## Case presentation

**Figure 1:**
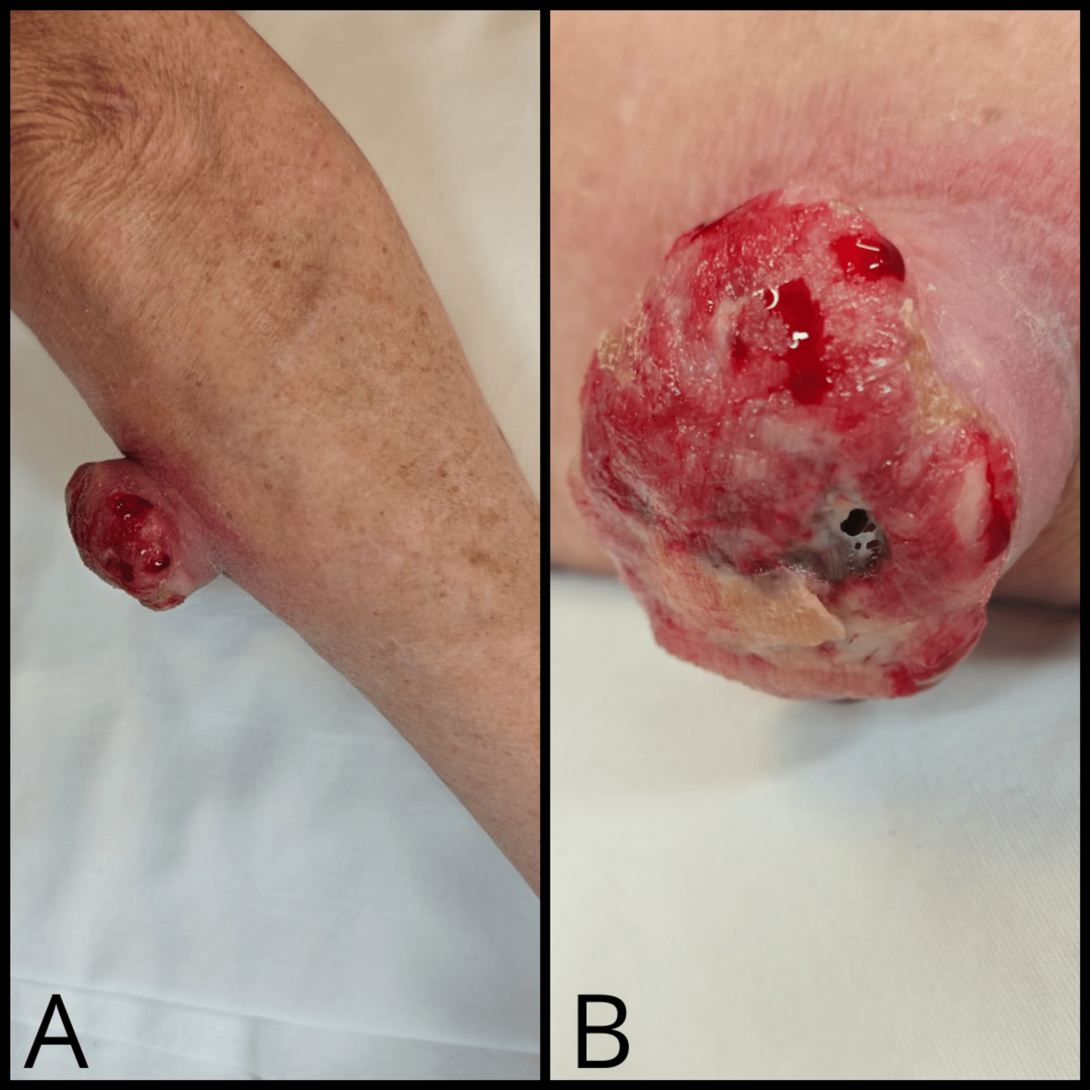
Exophytic lesion located on the right forearm near the elbow. (A) An ulcerated, 60 x 50 mm exophytic lesion can be seen. (B) A close-up of the lesion revealed well-defined edges, showcasing a compelling interplay of granulation tissue, areas of ulceration, and traces of bleeding.

A 70-year-old woman with type 2 diabetes and arterial hypertension presented with a one-year-old nodule on her right forearm. Physical examination revealed an asymptomatic, ulcerated, mobile, 60 x 50 mm exophytic lesion on her right forearm proximal to the elbow (Figure 1).

**Figure 2:**
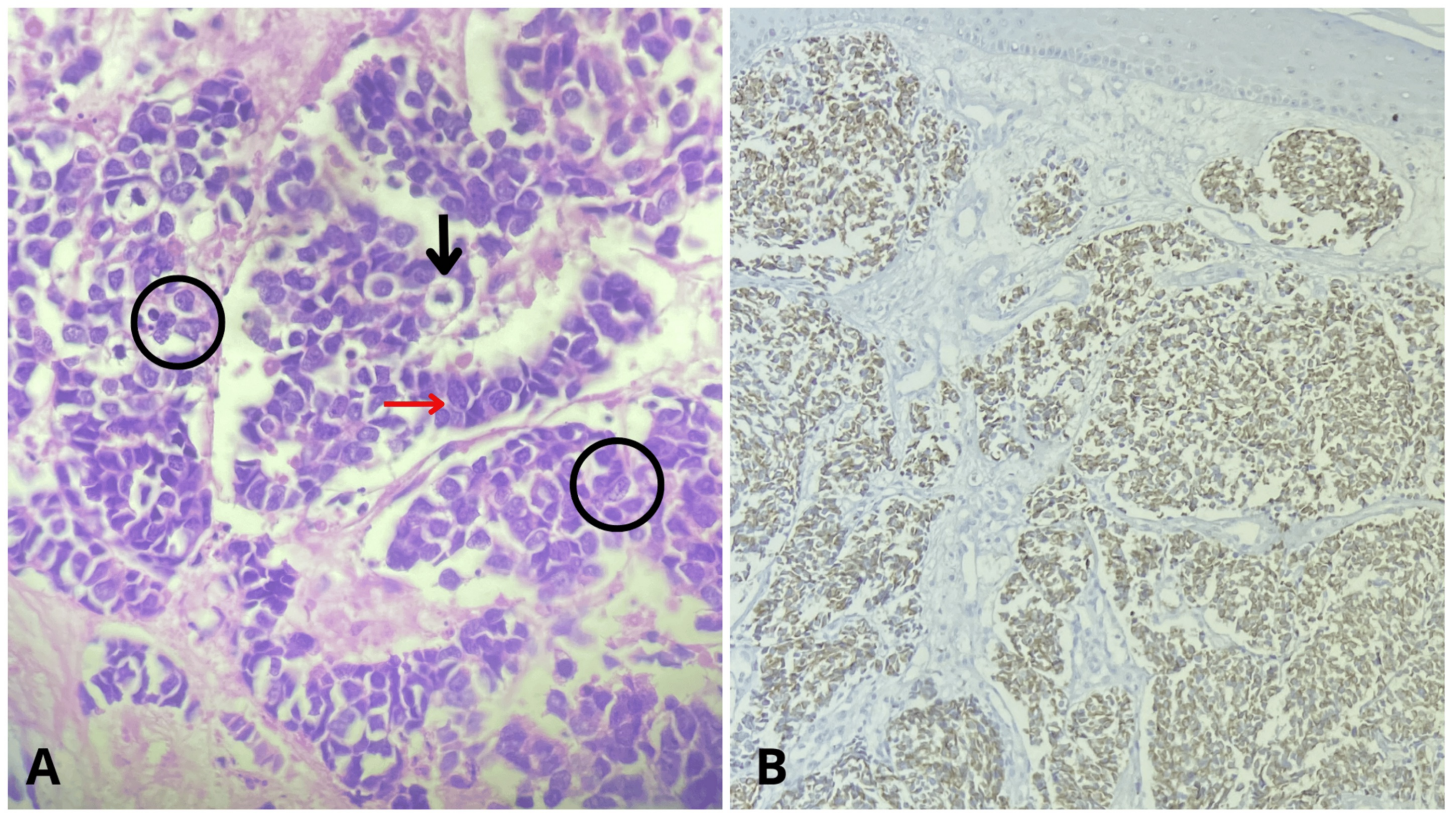
Biopsy findings of the exophytic lesion on the right forearm. (A) Hematoxylin and eosin staining revealed pleomorphic basophilic cells (indicated by the red arrow) with finely granular chromatin (circled in black), along with multiple abnormal mitoses (shown by the black arrow). Magnification: x40. (B) CK20 staining was focally positive, displaying a paranuclear dot-like pattern. Magnification: x40.

We observed a palpable lymphadenopathy in the ipsilateral epitrochlear region and a 2 cm lymph node in the axillary region, slightly indurated. Differential diagnoses were squamous cell carcinoma vs. amelanotic melanoma. The biopsy showed a neoplasm extending from the superficial to the deep dermis, consisting of lobular mantles surrounded by collagen. The cells were round, pleomorphic, basophilic, and had large nuclei with fine, granular chromatin, as well as multiple abnormal mitoses. Immunostaining for antibodies showed the following: CK20 was focally positive with a paranuclear dot-like pattern (Figure 2), chromogranin was focally positive, synaptophysin was negative, and CDX2 was negative.

The biopsy confirmed a diagnosis of ulcerated neuroendocrine carcinoma of the skin (MCC). We referred the patient with urgency to a national institute for extension studies and an interdisciplinary approach.

## Discussion

MCC has high rates of local recurrence and metastasis [1,4]. The case we present, a 70-year-old woman, is an illustrative example of how the different clues that guide us to include this entity in our approach can be integrated [6].

The oncogenesis of MCC is due to the integration of the Merkel cell polyomavirus into the genome or UV-mediated DNA damage secondary to chronic sunlight exposure [4,7]. It is important to mention that sunlight could also play a role in viral carcinogenesis through local immunosuppression. UV radiation may induce the expression of inflammatory mediators that alter the functions of antigen-presenting cells, leading to altered immune sensitivity and a decreased response to external stimuli [4]. Despite all the theories and advances aimed at elucidating the origin of carcinogenesis in MCC, the cellular origin remains unknown [1,4,7].

MCC presents as an erythematous nodule or violaceous plaque on sun-exposed skin, with the face being the most frequently affected site, followed by the neck and extremities. The nodule is usually firm, non-indurated, and grows rapidly, and can double in size within one to three months [1,4]. These clinical findings are not pathognomonic; a lesion exhibiting them raises suspicion for a range of other diseases; therefore, a biopsy is necessary for diagnosis [8].

This entity reveals tumor cells in the dermis and hypodermis with a salt-and-pepper chromatin pattern [9]. Diagnosis is confirmed by immunohistochemistry, with tumor cells expressing CK20, with a paranuclear dot pattern, as well as neuroendocrine markers like chromogranin A, synaptophysin, and CD56 [9]. The prognosis for this condition is poor when the tumor is >20 mm and regional lymphadenopathy is palpable [8].

Our patient was asymptomatic, had a rapid progression of the lesion, had an altered immunosuppression due to a long history of type 2 diabetes, was 70 years old, and the lesion was in an area considered to be under constant sun exposure. While these clinical characteristics are consistent with those reported in the literature [4,6,9], our case is notable for its large, exophytic lesion, lacking the shapes and colorations typically reported in the current literature. This unique presentation underscores the need for vigilance in diagnosing MCC, as it can manifest in unexpected ways. Given the aggressive nature of MCC, it is important to consider both its usual and unusual presentations.

## Conclusions

This case highlights the critical need to combine clinical and histopathological findings when dealing with complex differential diagnoses. Given the potential malignancy of this condition, it is essential to treat its evaluation as a medical emergency. Early intervention and prompt histopathological examination are vital for determining the prognosis.

## References

[1] Lewis DJ, Sobanko JF, Etzkorn JR (2023). Merkel cell carcinoma. Dermatol Clin.

[2] Toker C (1972). Trabecular carcinoma of the skin. Arch Dermatol.

[3] Sibley RK, Dehner LP, Rosai J (1985). Primary neuroendocrine (Merkel cell?) carcinoma of the skin. I. A clinicopathologic and ultrastructural study of 43 cases. Am J Surg Pathol.

[4] Becker JC, Stang A, DeCaprio JA, Cerroni L, Lebbé C, Veness M, Nghiem P (2017). Merkel cell carcinoma. Nat Rev Dis Primers.

[5] Paulson KG, Park SY, Vandeven NA (2018). Merkel cell carcinoma: current US incidence and projected increases based on changing demographics. J Am Acad Dermatol.

[6] Heath M, Jaimes N, Lemos B, Mostaghimi A, Wang LC, Peñas PF, Nghiem P (2008). Clinical characteristics of Merkel cell carcinoma at diagnosis in 195 patients: the AEIOU features. J Am Acad Dermatol.

[7] Prasad R, Katiyar SK (2017). Crosstalk among UV-induced inflammatory mediators, DNA damage and epigenetic regulators facilitates suppression of the immune system. Photochem Photobiol.

[8] (2024). National Comprehensive Cancer Network. NCCN Guidelines® Insights - Merkel cell carcinoma, version 1.2024. NCCN Clinical Practice Guidelines in Oncology: Merkel Cell Carcinoma. Version 1.2021.

[9] Walsh NM, Cerroni L (2021). Merkel cell carcinoma: a review. J Cutan Pathol.

